# The role of intraoperative monitoring in target selection in deep brain stimulation: A single centre study

**DOI:** 10.1016/j.prdoa.2025.100299

**Published:** 2025-01-06

**Authors:** Sandro Ibrulj, Dejan Georgiev, Žiga Samsa, Polona Mušič, Mitja Benedičič, Maja Trošt

**Affiliations:** a Department of Neurology, University Medical Centre Ljubljana, 1000 Ljubljana, Slovenia; b Faculty of Computer Science and Informatics, University of Ljubljana, 1000 Ljubljana, Slovenia; c Department of Neurosurgery, General Hospital Celje, 3000 Celje, Slovenia; d Department of Anaesthesiology, University Medical Centre Ljubljana, 1000 Ljubljana, Slovenia; e Department of Neurosurgery, University Medical Centre Ljubljana, 1000 Ljubljana, Slovenia; f Medical Faculty, University of Ljubljana, 1000 Ljubljana, Slovenia

**Keywords:** Deep brain stimulation, Intraoperative monitoring, asleep DBS

## Abstract

**Introduction:**

Intraoperative microelectrode recording (MER) and intraoperative test stimulation may provide vital information for optimal electrode placement and clinical outcome in movement disorders patients treated with deep brain stimulation (DBS). The aims of this retrospective study were to determine (i) how often the planned (imaging based) placements of electrodes were changed due to MER and intraoperative test stimulation in Parkinson’s disease (PD), dystonia and essential tremor (ET) patients; (ii) whether the frequency of repositioning changed over time; (iii) whether patients’ age or disease duration (in PD) influenced the frequency of repositioning.

**Methods:**

Data on the planned and the final placement of 141 electrodes in 72 consecutive DBS treated patients (52 PD, 11 dystonia, 9 ET) was collected over the first 8 years of DBS implementation in a single center. An association between the rate of electrode repositioning and the patients’ age, disease duration and the time/year of implementation was explored.

**Results:**

Analysis of all targets showed a change in final electrode placement in 39.7 % (56/141); 39.8 % (41/103) in PD, 40.9 % (9/22) in dystonia and 37.5 % (6/16) in ET. Annual analysis showed a decrease in rate of repositioning between the centre’s first and eighth year (p = 0.013) of implementation. No correlation was found between electrode repositioning rate and patient age (p = 0.42) nor disease duration (p = 0.09) in PD.

**Conclusion:**

This retrospective analysis confirms the benefit of MER and intraoperative test stimulation during DBS surgery in determining the final electrode position during the early / initial period of implementing the procedure. Our findings show a learning curve in successful preoperative planning in our centre achieved through experience.

## 1 Introduction

Deep brain stimulation (DBS) is a well-established and efficacious neurosurgical procedure for the alleviation of symptoms in a variety of movement disorders [1]. The beneficial clinical effect of DBS is dependent on various clinical and technical features, among which optimal electrode placement in the targeted structure is crucial [2]. The usual targets are the subthalamic nucleus (STN) in Parkinson’s disease (PD), the globus pallidus internus (GPi) in dystonia and the ventral intermediate nucleus of the thalamus (Vim) in essential tremor (ET) patients.

The traditional DBS operative technique is “awake” DBS, which involves intraoperative microelectrode recording (MER) and intraoperative test stimulation with the patient awake and participating. Preoperatively, atlas coordinates are applied to brain images to visualize the proposed target [3]. MER and intraoperative test stimulation contribute to the final adjustments of electrode position by recording electrophysiological signals and evaluating clinical effects on various stimulation planes [4], [5]. It has been shown that MER and intraoperative test stimulation provide valuable information that leads to electrode position readjustment in 20–40 % of cases [6], [7], [8]. However, recent advances in structural MRI imaging for target visualization, preoperative planning and electrode placement confirmation have allowed an emergence of a targeting approach without MER and intraoperative test stimulation with the patient kept under general anaesthesia – the so called “asleep” DBS [9].

The aims of this retrospective study were to determine (i) how often the preoperatively planned target for electrode placement was changed after MER and intraoperative test stimulation in DBS patients with PD, dystonia and ET; (ii) whether the frequency of replacement changed over time; and (iii) whether the replacement was influenced by patients’ age or (iv) disease duration in PD patients.

## 2 Methods

### 2.1 Patient selection and data collection

In this study we analysed the final placements of 141 implanted electrodes in 72 patients treated with DBS and compared them with the preoperatively planned placements.

We collected data on planned electrode positions based on preoperative MRI imaging and atlas coordinates, and compared them with the final electrode positions determined by additional intraoperative MER and intraoperative test stimulation in 72 consecutive patients treated with DBS for PD (52 patients), dystonia (11 patients) and ET (9 patients) at the University medical centre in Ljubljana (UMCL) between April 2015 and January 2023. DBS surgeries at the UMCL were first initiated in 2014.

Patients were referred to DBS surgery by a movement disorders specialist in accordance with established clinical recommendations and standard practices for patient selection in PD [10], ET [11] and dystonia [12].

### 2.2 Preoperative target selection

Preoperatively, high-resolution MRI images were merged with CT scans using Brainlab software and used to determine anatomical targets for electrode placement in each patient. An MRI (GE Signa Excite 1.5 T HDXT, 2002) using standard sequences (T2 TSE, T1 MP-RAGE, T1 IR) was performed one to three months before surgery and a CT on the day of surgery with the stereotactic frame in place. The target nucleus for all patients with PD was the STN, the GPi for all patients with dystonia and the ViM for all patients with ET. Direct target visualisation on MRI was used and supplemented with standard atlas coordinates to determine precise locations for STN and GPi. The standard target coordinates used for the STN were: 12 mm lateral to the midplane of the third ventricle, 2 mm posterior to the midcommissural point (MCP), and 4 mm below the intercommissural line (ICL). The target coordinates for the GPi were: 21 mm lateral to the midplane, 2 mm anterior to the MCP, and 5 mm below the ICL. Adjustments were made to avoid optical tracts as well as cortical and subcortical vascular structures if needed.

VIM was targeted based on standardized atlas coordinates using the intercommisural plane and midcommisural line as reference. Adjustments were made according to individual patient volume loss and atrophy, as well as the third ventricle size [13].

The coordinates for all anatomical targets were determined by the same two operating neurosurgeons in unison.

### 2.3 Surgical procedure

All surgeries were performed by M.B. and Ž.S. at the University Medical Centre Ljubljana. MER and intraoperative test monitoring were performed by neurologists. In patients with PD and ET all antiparkinsonian and anti-tremor medication was stopped at least 12 h before surgery in order to allow evaluation in the OFF medication state.

The surgical procedure was performed under initial sedation with dexmedetomidine for all patients. After burr-hole placement and opening of the dura, microelectrodes were placed into guiding tubes and descended above the predetermined anatomical targets. In all cases intraoperative recording and stimulation was performed using the Inomed MER system. Intraoperative MER was performed in a stepwise manner at 1 mm increments starting at 10 mm above and ending at 5 mm below the target defined by preoperative assessment. Recordings were routinely made in 3 parallel channels, the central channel aiming at the preoperatively determined target and the two additional channels placed at a 2 mm distance either anteriorly, posteriorly, laterally or medially to the central channel. Recordings were interpreted by a neurologist experienced in electrophysiology to determine characteristic firing potentials for each target (STN, GPi, VIM). An optimal initial placement depth was chosen based on electrophysiological readings in accordance with standard guidelines [14], [15].

The patient’s sedation was reversed prior to MER in PD and ET and intraoperative test stimulation was performed for the 3 parallel channels at optimal depths determined by MER. Intraoperative test stimulation was then performed at 1 mm increments above and below the target defined by preoperative assessment, up to a maximum of + /- 3 mm, for all 3 parallel channels. During intraoperative test stimulation in PD and ET patients, a slow stepwise upward titration of voltage in increments of 0.5 mA was performed at each depth during constant frequency (130 Hz) and pulse width (90 microsec) settings. In PD and ET patients, occurrence of stimulation induced beneficial and side effects was closely monitored and noted with at each tested depth. In patients with dystonia, MER and intraoperative test stimulation was performed under total intravenous anesthesia (TIVA) using remifentanil and propofol during constant frequency (130 Hz) and pulse width (120 microsec) settings, and side effects were monitored. Final channel choice and electrode placement depth for the STN and VIM targets was based on optimal stimulation effect on symptom alleviation and favourable side effect profile. In patents with dystonia, the final electrode position was determined according to favourable side effect profile. All patients were implanted with Medtronic neurostimulator systems (Activa PC, RC and Percept PC). All implanted electrodes were Medtronic 3389 models.

Discordance between the preoperatively planned position based on imaging and the final electrode position based on MER and intraoperative test stimulation was noted when the non-central channel was chosen for the final placement of the electrode.

An implantable pulse generator (IPG) was placed subcutaneously in the infraclavicular region in all cases under general anaesthesia. Postoperative CTs were lastly performed within 24 h in all patients to confirm final electrode location and to rule out postoperative complications. Surgical complications were noted.

### 2.4 Data analysis

The statistical analyses were performed using statistical software IBM SPSS Version 26 (SPSS Statistics Version 26, IBM, Armonk, New York, USA). All compiled data was processed by means of descriptive statistics, the Kullback-Leibler divergence test, the Chi-square test and binary logistic regression. *P* < 0.05 was considered statistically significant.

The Chi-square and the Kullback-Leibler divergence test were used to evaluate the final channel placement frequency for all 141 anatomical targets. Patients' age and final channel choice association was assessed using the Kullback-Leibler test. The effect of patients' age on final channel choice was assessed using a binary logistic regression model. The Kullback-Leibler test was used to determine frequency of central channel choice for each annual period between 2015 and 2023. A graphic linear trendline for both central and non-central channel choice was plotted for the period between 2015 and 2023 using the formula y = mx + b in SPSS. The Chi-square test was used to assess association between disease duration and central channel choice in PD patients.

## 3 Results

132 patients were recruited for this retrospective analysis. Patients whose data on intraoperative monitoring was missing were excluded from analysis. Finally, 72 consecutive patients (27 women) were included, among whom 69 underwent bilateral electrode placement and 3 (1 PD; 2 ET) a unilateral one. 52 patients were treated for PD, 11 for dystonia and 9 for ET.

In total, 141 final electrode placement positions from 72 patients were collected and analysed. Patients' demographic and clinical data are shown in Table 1.

**Table 1:** Patient demographics and target distribution.

Variable	Male	Female	Total
Patient, n, %	45 (62,5)	27 (37,5)	72 (100)
Age, mean	60,1	62,9	61,1
Laterality, n (%)			
Unilateral	1 (33)	2 (67)	3 (100)
Bilateral	44 (63,8)	25 (36,2)	69 (100)
Target, n (%)			
STN	37 (71,2)	15 (28,8)	52 (100)
GPi	4 (36,4)	7 (63,6)	11 (100)
VIM	4 (44,4)	5 (55,6)	9 (100)

**Fig. 1:**
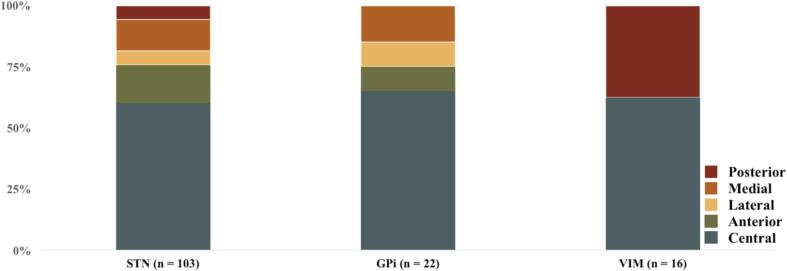
Choice of channel for final electrode positioning by individual target (STN = subthalamic nucleus; Gpi = globus pallidus internus; VIM = ventralis intermediate nucleus of thalamus).

The central channel was chosen for the placement of the permanent electrode in 60.3 % (85/141) of the targets. Among the non-centrally chosen channels, the anterior one was chosen in 12.8 % (18/141), the medial in 11.3 % (16/141) patients, the posterior channel in 9.9 % (14/141) and the lateral one in 5.7 % (8/141). There was no statistically significant difference among the selections of non-central channels. (Shown in Fig. 1) Separate subgroup analysis for each condition showed that the central channel was chosen more frequently in both PD (60.2 %), ET (62.5 %) and dystonia (59.1 %). There was no statistically significant difference among the selections of non-central channels in each subgroup (Shown in Fig. 1).

Patient’s age had no effect (p = 0.42) on the likelihood of central channel choice for final electrode position.

**Fig. 2:**
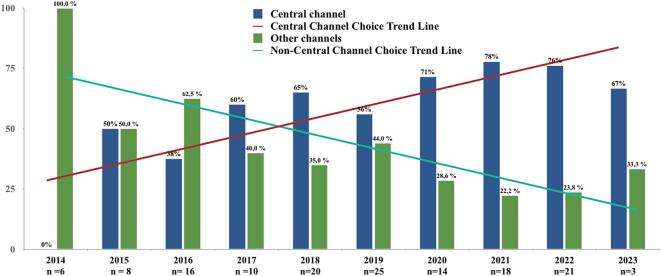
Final choice of channel for electrode placement, stratified by years of implementation. Annual analysis shows a significant increase in selection of central channel for final positioning between the centre's first year of DBS in 2014 and 2023 (p = 0.013). Bar graph linear trendline using y = mx + b is plotted for central channel choice (red line) and non-central channel choice (green line) trends. (For interpretation of the references to colour in this figure legend, the reader is referred to the web version of this article.)

An annual analysis of the implantations showed a statistically significant increase in the selection of the central channel for final electrode positioning each year between the centre's first year of DBS implementation in 2014 and 2023 (p = 0.013), with higher likelihood of choosing the central channel in recent years (shown in Fig. 2). A separate annual analysis for STN targets also showed a significant increase of central channel choice with time (p = 0.01).

No correlation was found between the electrode repositioning rate with disease duration (p = 0.09) in PD.

### 3.1 Complications

A small hemorrhage at the tip of the recording microelectrode in the right STN occurred in one PD patient after MER and before permanent electrode placement. The patient was left with mild left-sided hemiparesis.

## 4 Discussion

This retrospective analysis showed that using MER and intraoperative test stimulation during DBS implantation for the treatment of PD, dystonia and ET led to a change in final electrode position from the one planned preoperatively in 39.7 % of all cases. This was noted in all three patient groups and there was no significant frequency of choice of one other, non-central channel, for replacement. Patient age (p = 0.42) had no effect on final electrode placement, nor did disease duration in PD (p = 0.09).

Other studies have previously shown similar results with the final electrode position modification based on MER and intraoperative test stimulation in 20 to 40 % [16]. It was proposed that MER may consider the differences in an individual patient’s anatomy of the STN, GPi and ViM nuclei. It also allows a more optimal target border delineation as well as the evaluation of clinical effect during intraoperative test stimulation [17].

There is an ongoing debate regarding the added value of MER and intraoperative test stimulation in DBS surgery [18], [19]. Traditional awake DBS requires patient participation and limits the procedure to those who are tolerant to such surgery [20]. Patients with PD in particular can find this approach strenuous and stressful as the procedure is performed in the medication OFF state. Furthermore, patient participation ability can be affected by the interference of sedation. The awake procedure takes more time [21] and is more expensive [22]. MER is also associated with an increased risk of intracranial hemorrhage and other surgical complications in comparison to image-guided and image-verified approaches without MER [23], [24]. A recent comparative study however showed that MER did not increase the bleeding risk when a single trajectory and a guiding cannula were used [25]. Among our 72 patients, intraoperative intracerebral hemorrhage during MER occured in one (1.4 %), which is in line with other reports [26]. Anesthetics and sedatives used for surgery such as propofol and other GABAergic agents can affect neural activity, resulting in altered discharge properties and interference with electrophysiological recordings during MER. This effect potentially limits the ability to accurately identify the optimal target and subdomains using MER [27]. It has been reported that the anesthetic effect is type- and dose-dependent [27], [28]. However, with judicious selection of both, affected electrophysiological properties do not limit MER reliability [29]. In our centre, sedation with dexmedetomidine was reversed before MER and test stimulation in PD and ET patients. Residual effects of dexmedetomidine on neuronal activity cannot not be fully excluded, however recent studies have shown that it does not significantly affect MER or clinical outcome in STN DBS [30], [31].

On the other hand, “asleep DBS” is performed under general anaesthesia and optimal electrode placement in the targeted structures is ensured by direct visualisation of target on brain imaging [32]. MER is usually not performed. The electrode position is in such cases confirmed using intraoperative or postoperative MRI or CT [33], [34]. Precise preoperative target visualisation however depends on the availability of high-quality imaging and pre-operative targeting experience. Over the past years, advances in imaging quality such as high-field and volumetric high-resolution MRI imaging, as well as the developement of new sequences for both structure (T2*W, SWI, QSM, MDEFT, FGATIR etc.) and tract (DTI, TDI, MLFT etc.) delineation have improved direct visualization of targets [35], [36] and improved pre-operative planning [37], [38]. Pre-operative planning is also based on the assumption of rigidity of brain tissue, however it has been reported that a posterior brain shift of up to 4 mm may occur during DBS electrode implantation in deep brain structures including the targeted nuclei [39]. Recent reports have shown intraoperative MRI and CT as effective and precise in verifying the accuracy of final electrode position as MER [40], [41].

Several reports and studies have confirmed the accuracy of electrode placement in asleep DBS and shown equivalency of clinical effect on symptom alleviation of both techniques [42], [43]. However, direct comparative data is lacking, as there was a lot of heterogeneity in the used surgical techniques, imaging modalities, equipment, outcome measures and length of follow-up in these retrospective comparative studies on the two surgical approaches [44]. Nevertheless, one prospective randomized study was performed comparing both methods, and while clinical efficacy of both methods was shown to be equivalent, an increased incidence of stimulation-related long-term side-effects was described in the “asleep surgery” arm [45]. A similar finding was observed in another retrospective study [44].

In our centre we found that MER and intraoperative test stimulation provided important information that influenced final electrode placement, particularly in the earlier years of implementation. An annual analysis between our centre's earliest and most recent DBS implementation showed a statistically significant annual increase in the selection of the preoperatively planned target and lower frequency of change in final electrode placement after MER and intraoperative test stimulation for all targets (p = 0.013). A separate analysis for STN targets showed the same increase (p = 0.01). This finding indicates a learning curve of preoperative target planning. It is also in line with views of recent studies demonstrating the equivalency between awake and asleep DBS techniques when performed in experienced centres [46].

Limitations of this study include its retrospective study design and a relatively small study population. A few patients treated with DBS had to be excluded from analysis, due to the loss of results from intraoperative monitoring. Another limitation was grouping targets for annual analysis and correlation with age, however not enough GPi and VIM targets were available to be analysed separately in these conditions.

## 5 Conclusion

Several reports have recently shown equivalency of clinical effect and electrode placement precision between the so-called traditional “awake” DBS, which relies on MER and intraoperative monitoring, with “asleep” DBS, which relies on imaging alone. This retrospective analysis confirms the benefit of MER and intraoperative test stimulation during DBS surgery in determining the final electrode position during the early / initial period of implementing the procedure. Our findings show a learning curve in successful preoperative planning in our centre achieved through experience.

## Disclosures

This work was supported by The Slovenian Research and Innovation Agency (ARRS) through project J3-60059. The authors declare that there are no conflicts of interest relevant to this work. The authors declare that there are no additional disclosures to report.

## Ethical compliance statement

Chart review data was anonymized to protect PHI. Being a retrospective review, informed patient consent was not necessary for this work. We confirm that we have read the Journal’s position on issues involved in ethical publication and affirm that this work is consistent with those guidelines.

## CRediT authorship contribution statement

**Sandro Ibrulj:** Writing – review & editing, Writing – original draft, Project administration, Methodology, Investigation, Data curation, Conceptualization. **Dejan Georgiev:** Writing – review & editing. **Žiga Samsa:** Writing – review & editing. **Polona Mušič:** Writing – review & editing. **Mitja Benedičič:** Writing – review & editing. **Maja Trošt:** Writing – review & editing, Visualization, Supervision, Conceptualization.

## Declaration of competing interest

The authors declare that they have no known competing financial interests or personal relationships that could have appeared to influence the work reported in this paper.
